# Retrospective analysis of cohort risk factors and feeding phase timing associated with noninfectious heart disease deaths in U.S. feedlot cattle

**DOI:** 10.1093/tas/txab220

**Published:** 2021-11-24

**Authors:** Blaine T Johnson, David E Amrine, Robert L Larson, Robert L Weaber, Brad J White

**Affiliations:** 1 Beef Cattle Institute, Kansas State University, Manhattan, KS 66506, USA; 2 Department of Diagnostic Medicine and Pathobiology, Kansas State University, Manhattan, KS 66506, USA; 3 Department of Clinical Sciences, Kansas State University, Manhattan, KS 66506, USA; 4 Department of Animal Science and Industry, Kansas State University, Manhattan, KS 66506, USA

**Keywords:** brisket disease, cattle, congestive heart failure, feedlot, heart disease

## Abstract

Heart disease, specifically congestive heart failure, has become of increased interest to geneticists and cattle feeders. Data on cohort associations of risk factors related to heart disease and when heart disease deaths occur in U.S. feedlot cattle are limited. The study objectives were to 1) determine potential associations between feedlot cohort demographics and the risk of at least one noninfectious heart disease (NIHD) death occurrence and 2) determine potential association between feedlot cohort demographics and the timing of NIHD deaths during the feeding phase. Data were downloaded from commercial feedyard software and analyzed by constructing a generalized linear mixed model for both analyses. A binomial and Gaussian distributions for risk of NIHD death and timing of NIHD were utilized as link functions for their respective models. Our study population consisted of 28,950 cohorts (representing 4,596,205 cattle) that were placed in 22 U.S. commercial feedlots from January 01, 2016, to January 01, 2019. There were 3,282 cases of NIHD deaths from a population of 75,963 cattle that died during the 3-yr study period. Average cohort arrival weight’s effect on NIHD probability was influenced by arrival quarter and arrival year of placement (*P* < 0.01). Cohorts with steers were associated with a greater probability of at least one NIHD death (2.38%) compared with heifers (1.95%; *P* < 0.01). Increasing cohort size was associated with an increased probability of a cohort having at least one NIHD death (*P* < 0.01). The probability of a cohort having at least one NIHD death increased with increasing DOF categories from 1.51% in cattle fed 100 to 175 d, to 2.12% in cattle fed 176 to 250 d, and 2.87% for cattle fed 251 to 326 d. Cattle > 326 DOF were no different in the probability of a NIHD death compared with the other feeding categories. Timing of a NIHD death had a mean and median occurrence of 110 DOF with an interquartile range of 64 to 153 DOF. The effect of arrival weight on days at death was influenced by year placed with heavier cattle generally decreasing the model adjusted means of DOF at NIHD death. Arrival quarter was influenced by year placed on model adjusted means on the timing of a NIHD death. Steers with NIHD died later compared with heifers (*P* < 0.01) diagnosed with NIHD. In conclusion, multiple factors are associated with the probability and timing of a NIHD death. Probability of having at least one NIHD death within a cohort was low, and half of the deaths occurred before 110 DOF.

## INTRODUCTION

Heart disease, specifically congestive heart failure (CHF) or right heart failure (RHF), has been investigated recently (Neary et al., 2013, 2016; Heaton et al., 2019; Krafsur et al., 2019) in finishing cattle. Research has identified increases in cases classified as heart disease over time, suggesting that prevalence of heart disease may be increasing. Neary et al. (2016) reported that the risk of RHF from 2000 to 2012 doubled from 0.21 to 0.40 per 1,000 head placed. Some individual producers in the Western Plains had annual losses exceeding US $250,000 (Heaton et al., 2019). Previous research stated that animals that succumb to heart disease in the feedlot tended to die later in the feeding phase (Neary et al., 2015, 2016). Neary et al. (2016) found a median days on feed (DOF) for an RHF death was 132 d for Canadian cattle and 133 d for U.S. cattle. Due to the economic value of heart disease animals, including the cost of animal plus operational costs, and feed cost, there is a need to further investigate data to better understand heart disease risk and underlying factors that may influence heart disease. The purpose of this research is 2-fold 1) to determine the risk of at least one noninfectious heart disease (NIHD) death within a cohort and the associated cohort demographics that may influence risk of heart disease in the population and 2) to evaluate when a NIHD death occurs during the feeding period and evaluate cohort demographics that influence the timing of heart disease deaths.

## MATERIALS AND METHODS

Animal Care and Use Committee approval was not obtained for this study due to retrospective data being obtained from existing privatized databases from commercial feedyards.

### Data Source

Data used for analysis were under a confidential agreement with participating feedyards. Cohort and individual mortality data were collected from cattle placed in 22 commercial U.S. feedyards from January 1, 2016, to January 1, 2019, located throughout the mid and southern plains. After confirming that inclusion criteria were met, a total of 4,596,205 cattle were received comprising of 28,950 cohorts and 75,963 mortalities. A cohort is defined as a group of cattle that were acquired, managed, and marketed similarly but not necessarily commingled in the same physical location (pen) for the feeding phase. Data were exported from commercial feedyard systems and subsequently imported into R (R Core Team, 2020) for analysis.

### Risk of NIHD Cohort by Enrollment Criteria

**Figure 1.**
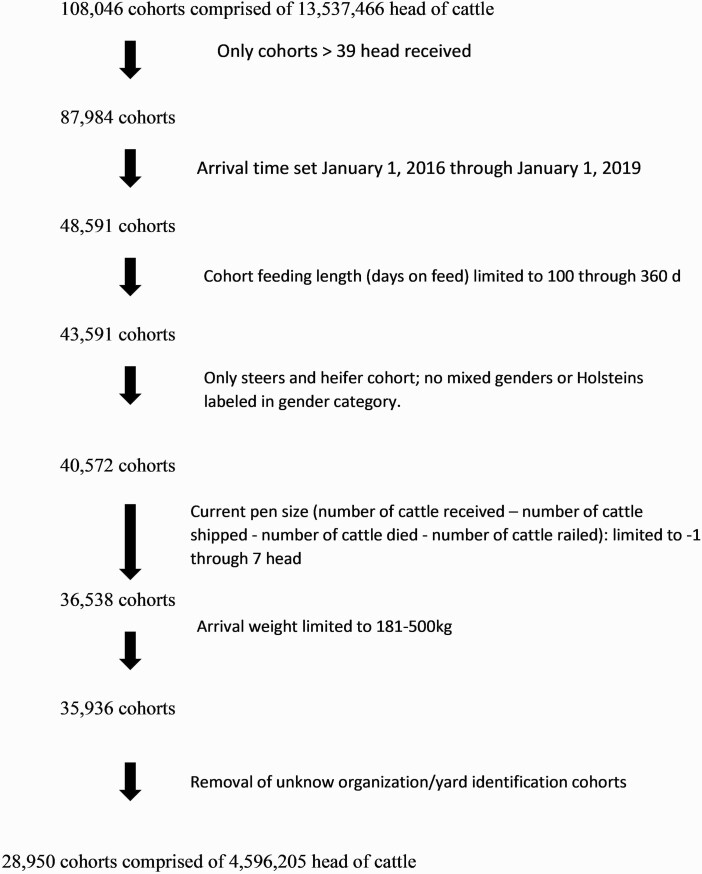
Data filtering from inclusion criteria applied to 22 U.S. feedyard data download to working dataset.

Enrolled cattle (Fig. 1) were categorized by animal identification, lot identification, average lot arrival weight (“in-weight”), arrival date to feedlot (“in-date”), death date (event date), DOF at death, sex, pen at death, number of cattle received in cohort, cause of death (“diagnosis”), and organization number (arbitrary identification number representing a unique feedyard). A unique identification number (UID) was created to distinguish animals within cohorts from their respective feedyard due to separate feedyards having similar cohort identification number systems. The UID was comprised of the organization identification number, feedyard identification number, and cohort identification number. Total DOF was calculated as ship date less arrival date (total DOF = ship date – arrival date). Estimated ship date was used when an actual ship date was not provided as the final date on feed for that lot in the total DOF calculation. If no known ship date or estimated ship date were recorded, then the observation was omitted from the dataset.

Included cohorts must have completed their respective feeding phase and be considered a closed cohort. Review of the data found multiple cohorts that had cattle remaining not shipped when they logically should have been (e.g., three cattle remaining in a cohort after multiple years since ship date). Thus, a new variable was created “current pen size” to evaluate for cohorts being closed. The following equation was used to calculate the parameters of current pen size: (Current Pen Size = cattle received – [cattle shipped + cattle railed + cattle dead] = 0). Cattle shipped is defined as cattle who finished their feeding period and were shipped to a harvest facility. Cattle railed is defined as cattle who were removed and not finishing their respective cohorts feeding period due to health-related reasons (i.e., muscular skeletal injury and lack of thriving). Data evaluation revealed that multiple cohorts remained with −1 or ≤ 7 cattle in current pen size when they should have been 0 due to shipment date and time to data download with no new entries. Thus, authors deemed data acceptable to include a current pen size range from −1 to 7 cattle as a “closed pen” to accommodate for a likely accounting error.

Cohort inclusion criteria included: greater than 39 cattle were placed, cohort with current pen size at close-out of −1 to 7, and average arrival weight was greater than or equal to 182 kg and less than or equal to 545 kg. Cohorts were limited to sex listed as heifer or steer. Other options under the sex category including mixed sex, bulls, or Holstein were not included. Cohorts must have been on feed at least 100 d before shipping to harvest. For cohorts that met the inclusion criteria, a placement quarter variable was created from the arrival month of placement. The following criteria were used to create the placement quarter variable: 1 = January through March, 2 = April through June, 3 = July through September, and 4 = October through December.

Individual mortality data used in this analysis were gathered from their respective feedyard health software. Multiple diagnosis categories were utilized to define specific diseases common in fed cattle. For the purpose of this paper, entered diagnoses were evaluated and condensed into five disease categories: acute/atypical interstitial pneumonia (AIP), bovine respiratory disease complex (BRD), gastrointestinal disease + bloat (GI + Bloat), NIHD, and other (any diagnosis that was not AIP, BRD, GI + Bloat, or NIHD).

### DOF Analysis of Heart Deaths

Cohorts meeting the inclusion criteria had their individual mortality data analyzed for when a NIHD death occurred during the feeding phase. A new variable was created and called “event days on feed.” This was calculated by using the following formula (Event DOF [days] = Death date – Arrival date). Individual deaths categorized for NIHD were included in the analysis. Infectious heart disease cases were removed from the heart disease category prior to analysis. Table 1 presents the diagnosis codes that comprised the heart disease category.

**Table 1.** Disease diagnosis categories incorporated into NIHD case definition

Congestive heart failure
HD heart disease
HEA heart failure
Heart disease
Heart failure
Heart failure left/right
Heart failure left/right
Heart problem
Left heart failure
Heart failure
Mechanical heart failure
Infectious heart failure
Congestive heart failure

### Statistical Analysis

#### Risk analysis for any NIHD death within a cohort.

A generalized linear mixed model was fit into R Studio (R Core Team, 2020) using the “lme4” package (Bates et al., 2015) and “glmer” function. Feedyard was included as a random intercept to account for lack of independence of cohorts within feedyard. A binomial outcome of 0 or 1 for heart death in lot was created, 0 = no NIHD deaths within the cohort and 1 = at least one NIHD death within cohort, for the outcome variable, and fixed effects included: arrival quarter, DOF, arrival weight, sex, arrival head received (lot size by quartiles), and arrival year. To avoid violating the assumption of linearity, the total received head in each lot was categorized into four equal groups using the dplyr::ntile function in R (Wickham et al., 2019). By using this method, there is the possibility of overlapping values in the groups; however, authors wanted to keep the groups as equal as possible with respect to the number of lots in each group. A univariate analysis was performed and only significant factors (*P* ≤ 0.05) were included. All factors of the model were found to be significant and a second model was created incorporating interactions. All possible 2-way interactions were incorporated and only significant 2-way interactions were included (*P* ≤ 0.05). The final risk model was created utilizing a backward elimination process (Dohoo et al., 2012), where all factors and interactions were included and then eliminated when found to be nonsignificant (*P* > 0.05).

#### DOF of heart death individual analysis.

Individual heart disease mortalities (*n* = 3,282) and their associated cohort data were exported from 19 U.S. commercial feedlots; 3 of the feedyards from the 22 used with the risk analysis reported no NIHD deaths and thus were not included in the NIHD DOF analysis. A generalized linear mixed model was fit in R Studio (R Core Team, 2020) using the “lme4” package (Bates et al., 2015) and “lmer” function to assess the association of head received, arrival quarter, gender, arrival weight, and arrival year on the outcome DOF at the time of a NIHD. Feedyard was included as a random intercept to account for the lack of independence within feedyards. The final model was created by initially including all covariates and interactions, and then a backward variable elimination process was used to remove nonsignificant interactions and covariates (*P* > 0.05).

## RESULTS

### Descriptive Statistics

**Figure 2.**
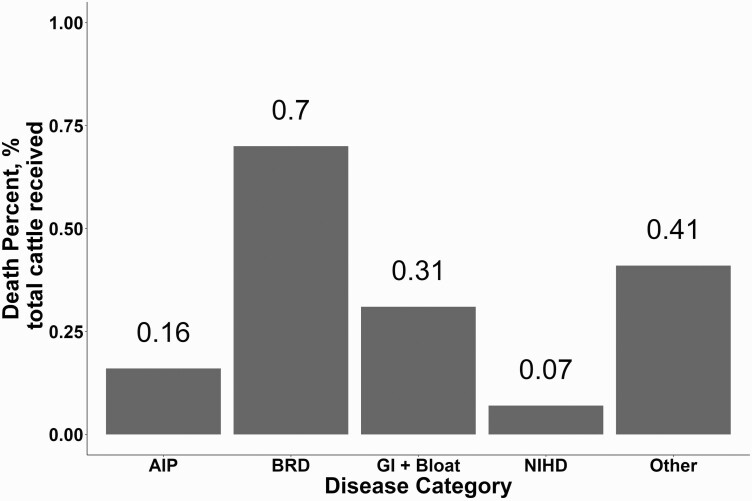
Percent of diagnosis per disease category expressed as percent of total cattle received. Abbreviations: AIP, atypical/acute interstitial pneumonia; BRD, bovine respiratory disease; GI + Bloat, gastrointestinal disorders and bloat; NIHD, noninfectious heart disease; Other, all other non-categorized diseases.

The final dataset consisted of 28,950 cohorts; 11,927 cohorts were heifers and 17,027 were steers. The total cattle received during the 3-yr observational study was 4,596,205. Figure 2 shows the total cattle received to the feedyards which died (*n* = 75,963 cattle) during the feeding phase of various diseases expressed as a percent of total cattle received. Death loss for NIHD was 0.07% or a 7 in 10,000 cattle across all 22 feedyards over the 3-yr period. This number was calculated by total NIHD deaths divided by total cattle received (3,282 cattle/4,596,205 cattle). There were 457 NIHD deaths in 2016 out of a population of 1,081,903 cattle accounting for 0.04% or 4 in 10,000 cattle. In 2017, NIHD accounted for 1,283 deaths out of a population of 1,637,876 cattle accounting for 0.078% or 8 in 10,000 hd of cattle. Finally, in 2018, 1,542 cattle died due to NIHD in a population of 1,876,426 cattle representing 0.08% (8 in 10,000 cattle). NIHD deaths accounted for approximately 4% of the total dead population (3,282/75,963). Variables significantly (*P* < 0.05) associated with the probability of having at least one NIHD death in a cohort included sex, cohort size, and cohort feeding length (DOF) and the 2-way interactions between arrival weight and arrival year, arrival weight and quarter of placement. Variables significantly (*P* < 0.05) associated with influencing timing of NIHD death include sex and cohort size, and the 2-way interactions between arrival year and arrival quarter, arrival year and arrival cohort average weight.

### Association of Cohort Risk of At Least One Heart Death Within Cohort

**Figure 3.**
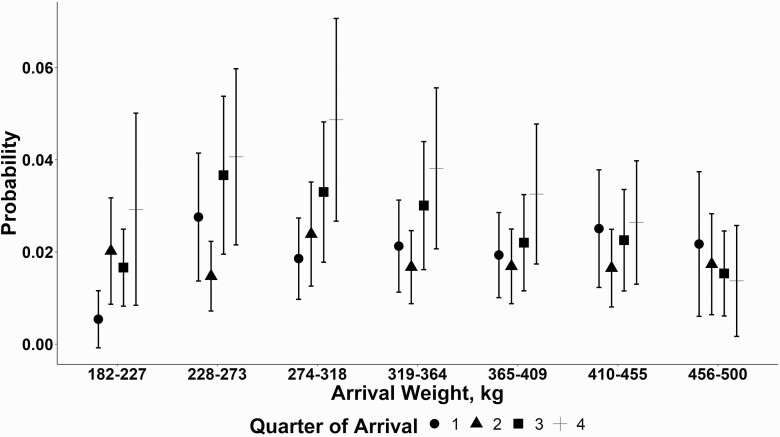
Model estimated means with one standard error probability of at least one NIHD death by cohort arrival weight (x-axis) and cohort arrival quarter (shape).

**Figure 4.**
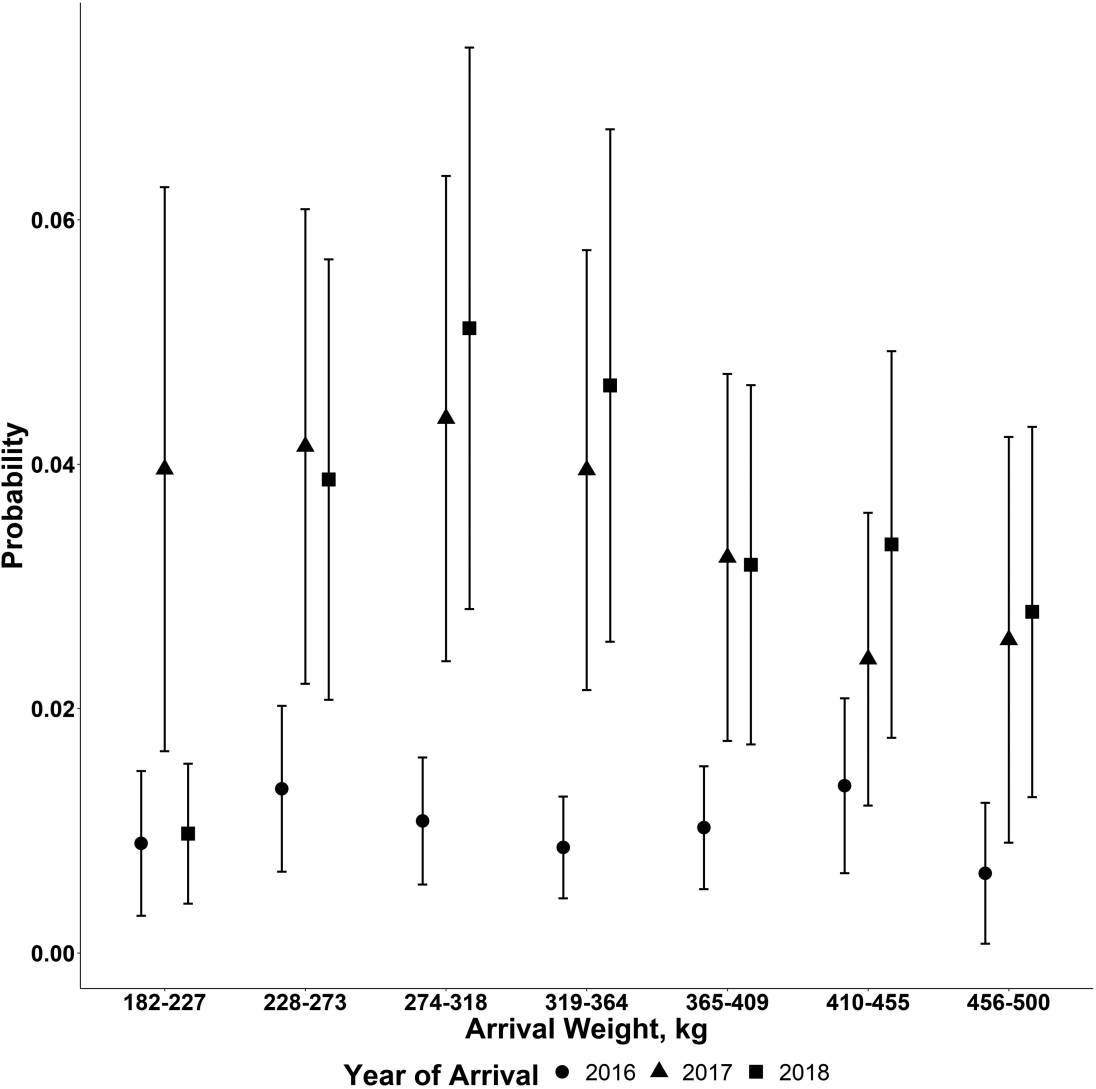
Model estimated means with one standard error of at least one NIHD death by cohort arrival weight influenced by cohort arrival year (shape).

The effect of cohort weight on the probability of a cohort having at least one NIHD death was influenced by cohort arrival quarter and year of placement. The association of arrival weight category with the risk of having a cohort with at least one NIHD death generally decreased with heavier weights influenced by arrival quarter (Fig. 3). Cattle placed in the second quarter had the same risk of NIHD death across all weight groups. The greatest risk of having a NIHD death within a cohort was found in the 273 to 318 kg cohorts placed in the fourth quarter compared with all other cattle placed at all other quarters. Cohorts placed in the first quarter at 182 to 227 kg had the lowest risk (0.005% probability). However, the lighter-weight cattle in our study (182 to 227 kg) did not differ in the risk from the heavier placed cattle (445 to 500 kg). The association of arrival weight with the probability of at least one NIHD death in a cohort was influenced by placement year (Fig. 4). In 2016, all placement weight categories had no differences in cohort probability of a NIHD death. Cattle placed in 2017 and 2018 had no differences in probabilities of a NIHD death across weight category except for 2018, and 182 to 227 kg placed cattle had a lower probability (0.009, lower confidence limit [LCL] 0.003; upper confidence limit (UCL) 0.02) of a NIHD death within cohort compared with 2017 (0.037, LCL 0.012; 0.11 UCL).

Additionally, steer cohorts were associated with having a greater model estimated means probability of a NIHD death compared with heifers (2.38% [LCL 1.0%, UCL 5.8%] vs. 1.95% [LCL 0.78%, UCL 4.7%], respectively) (*P* < 0.01). The model estimated means probability of NIHD increased linearly with cohort size quartile from the lowest probability of 1.12% in quartile 1 to 3.82% in quartile 4. Quartile 4 was not different from quartile 1 but was different than quartiles 2 and 3 (1.93% and 2.57%, respectively).

The model estimated means probability of at least one NIHD death in a cohort increased with increasing DOF for cohorts from 1.51% to 2.87% for cattle fed 100 to 175 and 251 to 326 d, respectively. Model estimated means estimate probability decreased to 2.33% in cattle that were fed > 326 d, which was not (*P* = 0.90, *P* = 0.9985, *P* = 0. 99) different compared with the 100 to 175, 176 to 250, and 251 to 326 categories of DOF, respectively.

### Timing of NIHD Death Modeled Results

Variables significantly (*P* < 0.05) associated with the DOF of NIHD death in the final model were cohort size and sex and the 2-way interactions of arrival weight and arrival year, and arrival year and arrival quarter.

**Figure 5.**
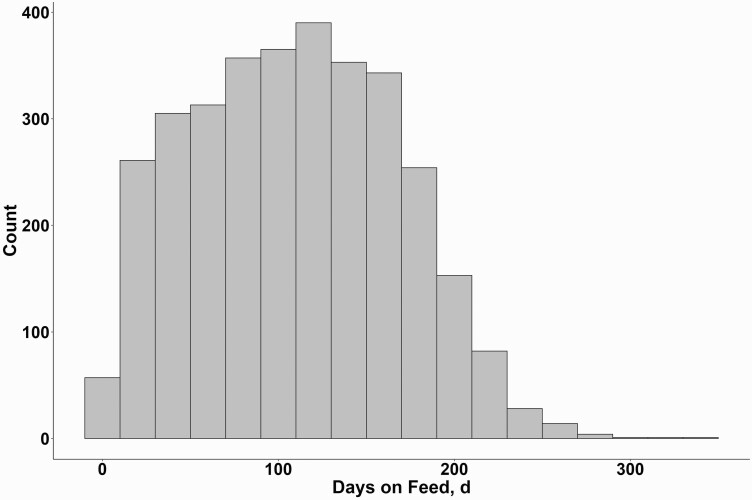
Count of NIHD deaths by DOF at the time of death (mean and median = 110 d); totaling 3,282 NIHD deaths observed over 3 yr from 19 U.S. feedyards.

**Figure 6.**
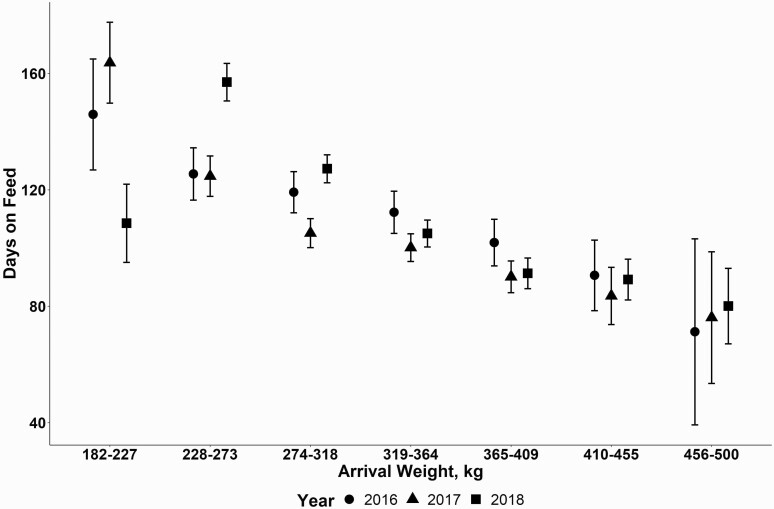
Model estimated mean estimates with one standard error of a NIHD death event (DOF) affected by average cohort arrival weight influenced by arrival year (shape).

**Figure 7.**
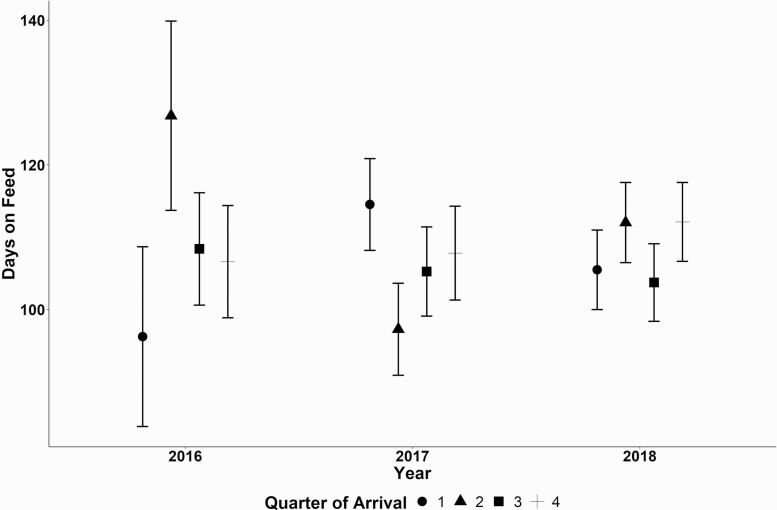
Model estimated mean estimates with one standard error of a NIHD death event (DOF) affected by cohort arrival year influenced by arrival quarter (shape).

Cattle that died from NIHD died on average at 110 DOF (Fig. 5). The median DOF death was also found to be 110 d. The interquartile range of a NIHD death occurred between 64 and 153 DOF in this study. Cattle placed at heavier weights tended to have a lower model estimated DOF at NIHD death (Fig. 6) with the effect being influenced by placement year. Placement quarter’s effect of when a NIHD death occurs was also influenced by placement year (Fig. 7). The effect of sex found that steers died later compared to heifers at 114 d ± 4 d compared to heifers which died at 102 ± 5 d (P < 0.01). Finally, cattle placed from cohorts’ sizes of 60 to 101 cattle died approximately 8 d earlier (LCL 91 d, UCL 111 d) than other placement groups (*P* < 0.01).

## DISCUSSION

A problem with operational feedyard data is a lack of consistent standardized reporting of data across feedyards (Corbin and Griffin, 2006). Our retrospective data include many case definitions of NIHD, which can be specific to a feedyard and/or organization and potentially differ among trained personnel. Each feedyard entered a diagnosis for health data for their respective system for cause of death. To accommodate the inconsistent case definition of what defined heart disease, our study case definition included all noninfectious diseases that affected the heart which was labeled the primary cause of death.

Previous literature has described the individual risk of RHF and factors that affect risk of RHF in Canadian and U.S. feedlot cattle (Neary et al., 2016). Bassett (2019) performed an epidemiological investigation on CHF of one feedyard in western Nebraska over 6 yr, which looked at number of CHF cases per 100,000 cattle head days and the timing of CHF diseases during the feeding period. The current study evaluated potential associations of cohort factors on the probability of at least one NIHD death and the timing of NIHD during the finishing phase. Our data showed that the risk of NIHD death doubled from 4 per 10,000 cattle received in 2016 to 8 per 10,000 cattle received in 2017 and 2018. Differences from 2016 compared with 2017 and 2018 could be due to more contributing feedyard data during 2017 and 2018, cattle type procured at each respective feedyard, or other unknown reasons. Neary et al. (2016) estimated the incidence of RHF in cattle to be between 4 and 17 per 10,000 cattle entering U.S. feedyards and reported an increase in mean risk per 1,000 Canadian cattle for RHF from 0.21 in 2000 to 0.4 in 2012. Bassett (2019) found that the proportion of cases of CHF doubled from 2011 to 2016. One concern with operational feedlot data is the accuracy of which the data are measured. Theurer et al. (2015) stated that accuracy of measured health outcomes is a potential source of differential error. The question of whether NIHD deaths are increasing or are veterinarians and feedlot personnel getting better at diagnosing them is still a valid confounding concern. Albeit, the data presented here saw increases in the number of cases throughout multiple different time periods and locations. 

The effect of arrival weight on the probability of a NIHD death was influenced by quarter of feedyard placement in our data. Average cohort placement weight had the numerically lowest model estimated probability of a NIHD death in the 182 to 227 kg groups, whereas the 273 to 318 kg average cohort placement weights had a numerically greater probability of a heart death when placed in the fourth quarter compared with other placement weights and placement quarters. Cattle placed in the second quarter had a similar probability of a NIHD death across all weight classes. Lighter-weight cattle had a similar probability of a NIHD death to cattle placed at 445 to 500 kg. Neary et al. (2016) reported that increasing weights at feedyard placement did not increase the risk of an RHF, with the assumption more weight to gain means more DOF. Bassett (2019) evaluated cohort percent finish, which was defined as “Degree of Finish (% Finish) = DOF at diagnosis/(lot end date – lot start date),” at the time of individual CHF death and reported that there was potential for clustering, but CHF was distributed throughout the feeding period and heavily skewed with increased deaths in greater percent finished cohorts. “Degree of finish” variable was designed to be a representation of an approximate degree of fattening with respect of timing to their respective cohort at the time of death. Our data found that that lighter-weight (181 to 227 kg) cattle had a relatively similar probability of a heart disease death within cohort as heavier placed cattle (409 to 455 kg). In addition, our data also found that weight was also affected by the year of placement into feedyard. In 2016, our data saw a numerically lower probability of a heart disease death across all weight classes compared with 2017 and 2018 data. Neary et al. (2016) found year-to-year variation in RHF with an overall 2-fold increase in risk from 2000 to 2012. Bassett (2019) evaluated CHF in one feedyard over 6 yr and saw an increased number of cases per 100,000 cattle head days from 2011 through 2016. Our data showed a numeric increase in a probability of at least one NIHD death within a cohort from 2016 to 2018, suggesting that some potential factor (i.e., cattle type) within year is influencing cattle dying of NIHD.

Steer cohorts had a greater probability of a cohort having at least one NIHD death compared with heifers (*P* < 0.01). Neary et al. (2016) found that steers in the United States had 39% greater odds of RHF compared with heifers, but no differences were observed in Canadian cattle sexes. Our data showed that steers had a 22% (2.38% vs. 1.95%) greater probability of at least one NIHD death compared with heifers diagnosed with NIHD. A field investigation by Moxley et al. (2019) found an overrepresented proportion of steers (16) compared to heifers (1) dying of heart disease over a 15-month period at one feedyard.

It was hypothesized that increasing heavier finishing weight increases heart failure risk by increasing fat deposits around the cardiopulmonary structures (Krafsur et al., 2019). This would suggest that there would be a longer average time from arrival at the feedyard to death due to NIHD. Our data found that median time from arrival to NIHD death (110 d) was shorter compared with previous literature (Neary et al., 2016) at 133 d. Our observation of near-normal distribution of time from feedlot arrival to at least one death due to NIHD reinforces the finding that NIHD was not found to increase over the feeding phase. Bassett (2019) reported a relatively normal distribution of percent finished at time of death due to CHF, meaning that approximately one-half of the cases happened before the cohort was 50% to 60% finished. These data suggest that NIHD is not necessarily a late-day disease in finishing cattle. However, our analysis found that increasing DOF for a cohort was associated with an increased probability of having at least one NIHD death within the cohort from 100 to 326 d finishing periods. There was an increase in model estimated mean cohort probabilities of a NIHD death from 100 (1.51%) to 326 (2.87%) DOF. Feeding greater than 326 d showed no evidence of being different from other DOF categories with a model estimated mean probability of 2.33%, meaning that risk was no different in cattle fed for a considerable length of time compared with cattle earlier in the finishing period. These data are consistent with the study of Neary et al. (2016) and Bassett (2019), in which, respectively, they found CHF throughout the feeding period. Furthermore, we found that half of the NIHD deaths occurred before 110 d. Neary et al. (2016) found half of their heart disease deaths occurring after 19 wk (>133 d) on feed. Bassett (2019) showed that CHF cattle died in the greatest proportion between 121 and 181 d after arrival to the feedyard. Thus, assessment of the timing of NIHD deaths across multiple studies has shown that cattle dying from a heart disease event are not strictly dying during the later days of the feeding period where heavier and more fatten cattle would be represented.

In addition, the current study looked at average cohort arrival weight’s effect on the timing of NIHD death. Arrival weight category was significantly associated with the timing of NIHD death with heavier weights having a general trend of lower DOF when they died. Heavier placed cattle had a model estimated means DOF that was numerically lower than lighter placed cattle; however, there was considerable variation in the point estimate. This was likely due to having fewer cattle placed in heavier weight categories and consequently fed for a shorter period. However, one could argue that they are not at risk for the same amount of time during the feeding period compared with a lighter (282 to 227 kg) placed cattle.

The results of this study indicated that multiple factors were associated with increased probability of having at least one NIHD death within a cohort. The current study’s investigation found that interactions of the studied cohort factors have a role in the biology of risk of NIHD. Additionally, the association of timing of a NIHD death was found to have a near-normal distribution throughout the feeding period with half of the deaths occurring before 110 d. Overall, the risk associated with NIHD was very low in the current study, and several factors were found to be associated with the probability of at least one NIHD death within a cohort. However, the probability of a cohort having at least one NIHD death was relatively minimal.

Even at a low prevalence, heart disease in feedlot cattle remains troubling due to animals dying throughout the feeding phase, the financial impact of NIHD, and the lack of a clear causal pathway.

## LIMITATIONS

In observational studies, there tends to be a lack of consistent, standardized reporting of data across feedyards (Corbin and Griffin, 2006). In our retrospective study, certain limitations need to be addressed. One is that our study was conducted on data that were entered from multiple feedyards having different personnel diagnose the diseased animals at necropsy with various training backgrounds. As previously stated, case definition varied between yards (Table 1), which carries the potential of misclassification bias. To help control some of this bias, multiple heart disease categories were lumped into one category of NIHD. Additionally, the risk factors evaluated were not randomly dispersed among cohorts, as with all observational studies, and were subjected to unknown confounding factors. To help address bias and confounding, our analysis was set up as a multivariate mixed effects and had feedyard set as a random intercept. Thus, the results and conclusions are limited to the data presented and the cattle that fit our inclusion criteria.
